# Developments in proton MR spectroscopic imaging of prostate cancer

**DOI:** 10.1007/s10334-022-01011-9

**Published:** 2022-04-20

**Authors:** Angeliki Stamatelatou, Tom W. J. Scheenen, Arend Heerschap

**Affiliations:** Department of Medical Imaging (766), Radboud University Medical Center Nijmegen, Geert Grooteplein 10, P.O. Box 9101, 6500 HB Nijmegen, The Netherlands

**Keywords:** Prostate cancer, Proton magnetic resonance spectroscopy, State-of-the-art review, Spectroscopic imaging

## Abstract

In this paper, we review the developments of ^1^H-MR spectroscopic imaging (MRSI) methods designed to investigate prostate cancer, covering key aspects such as specific hardware, dedicated pulse sequences for data acquisition and data processing and quantification techniques. Emphasis is given to recent advancements in MRSI methodologies, as well as future developments, which can lead to overcome difficulties associated with commonly employed MRSI approaches applied in clinical routine. This includes the replacement of standard PRESS sequences for volume selection, which we identified as inadequate for clinical applications, by sLASER sequences and implementation of ^1^H MRSI without water signal suppression. These may enable a new evaluation of the complementary role and significance of MRSI in prostate cancer management.

## Introduction

Prostate cancer (PCa) is the second most commonly occurring cancer in men and the fifth leading cause of cancer death, with an estimated 1.4 million new cases and 375,000 deaths worldwide in 2020. Relatively little is known about the etiology of PCa, however age, family history, and genetic mutations are established risk factors [1]. Since the 90’s, prostate cancer mortality rates are declining in most countries with a high level of medical care [2, 3]. This is attributed to advancements in treatment and earlier detection through screening [4, 5]. In particular, prostate-specific antigen (PSA) testing allows early cancer detection and significantly affected mortality rates [6].

Autopsy studies of men not diagnosed with PCa have shown a PCa incidence of 60% in men over 80 years old [7], so screening for the disease with PSA testing also finds many cancers that would probably never need any treatment. Therefore, a major issue in PCa management is to distinguish between potentially aggressive cancers that are clinically significant requiring treatment and those that will not need immediate treatment [8]. In histopathology of biopsies, the aggressiveness of tumor lesions is characterized by Gleason grades on a scale from 1 to 5, determined at two locations, which are combined in a Gleason score (GS). Often lesions with a GS ≤ 3+3 are defined as low risk, with 3+4 as intermediate and with ≥ 4+3 as high risk. To better connect with clinical practice GSs are regrouped in Grade groups (GrG), i.e., GrG1=GS≤6, GrG2=GS3+4, GrG3=GS4+3, GrG4=GS8, GrG5=GS9-10 [9, 10].

The standard way to confirm the presence and nature of cancer in the prostate is transrectal ultrasound (TRUS)-guided biopsy specimens, analyzed by histopathology [8]. More recently, multi-parametric MRI (mpMRI) and MRI-guided targeted biopsy [11] have emerged as important tools in the detection, grading and staging of PCa [12–14]. The Prostate Imaging Reporting Αnd Data System (PI-RADS) [13] aims at uniform reading of mpMRI in a structured reporting system assessing the likelihood of disease with clinical significance, using a combination of T2-weighted MRI (T2w-MRI), diffusion-weighted MRI (DWI) and dynamic contrast-enhanced MRI (DCE-MRI). In PI-RADS, the assessment by DCE is secondary to T2W and DWI. Recently, the necessity to use DCE is further disputed and using an endorectal coil at 3T is not advocated anymore [15]. In this way, the detection, localization, characterization, and risk stratification of tumors in patients suspected for PCa are improved [12, 16].

While MRI parameters can assess anatomical, morphological and some physiological abnormalities associated with cancer development, complementary information on molecular aspects of this development can be derived from metabolic readouts, of which some may underlie earlier or more specific phases of disease progression. Tissue metabolites can be assessed non-invasively by ^1^H Magnetic Resonance Spectroscopic Imaging (MRSI). In ^1^H MRSI of prostate tissue a number of signals of metabolite protons, including those in citrate, choline compounds, spermine and creatine are detected. Ratios of these signals can serve as biomarkers in the detection, localization and characterization of PCa. As ^1^H MRSI can be added, essentially seamlessly, to MRI procedures it may reinforce mpMRI in the non-invasive diagnosis of PCa [17–20]. In particular as mpMRI currently suffers from a low pooled specificity [21] and low inter-reader reproducibility [22].

Although MRSI was part of the first PI-RADS version, it was decided not to include it in later versions, i.e., PI-RADS 2.0 [23]. This decision was made because at that time the technique was less robust than the MRI methods and therefore difficult to apply successfully in clinical routine, in particular if no significant in-house expertise was available. Initially, it also required rather long examination times and lacked standardized automated processing and adequate data display [17]. Application of the technique is also muted by the still often encountered believe that an endorectal coil is an absolute requirement for MRSI of the prostate, while most MRI is now performed without such a coil.

Since its introduction, significant progress has been made in the development of prostate ^1^H MRSI. This progress made it possible to acquire 3T MR spectra of voxels with effective sizes down to 0.3–0.6 cm^3^ with sufficient signal-to-noise (SNR) and spectroscopic resolution to detect metabolites of interest in measurement times below 10 min [17, 20].

MRSI of the prostate has been reviewed most recently by Tayari et al. [17], Kurhanewicz et al. [19], and Kobus et al. [24]. After these reviews some new developments concerning prostate MRSI with sLASER without an endorectal coil, prostate MRSI without water signal suppression and MRSI reconstruction have been presented. Furthermore, several new approaches improving MRSI of the brain have been reported that are promising for application to the prostate as well. For overviews including the application of other MRS methods to prostate cancer see Sharma et al. [25] and Jagannathan [26].

In this paper, we review recent developments of ^1^H MRSI applied to prostate cancer, covering topics such as specific hardware, dedicated acquisition sequences and processing and quantification techniques. Emphasis will be given to advancements in MRSI methods that may overcome difficulties currently encountered in routine clinical MRSI applications, enabling a new evaluation of the complementary role and significance of MRSI for PCa management.

## ^1^H MRSI of the prostate

Most clinical applications of MRSI employ the ^1^H nucleus, because it is abundant in body compounds, has a relatively high sensitivity and the required MR hardware is widely available in the clinic.

### ^1^H MR visible metabolites in the human prostate in vivo

**Fig. 1:**
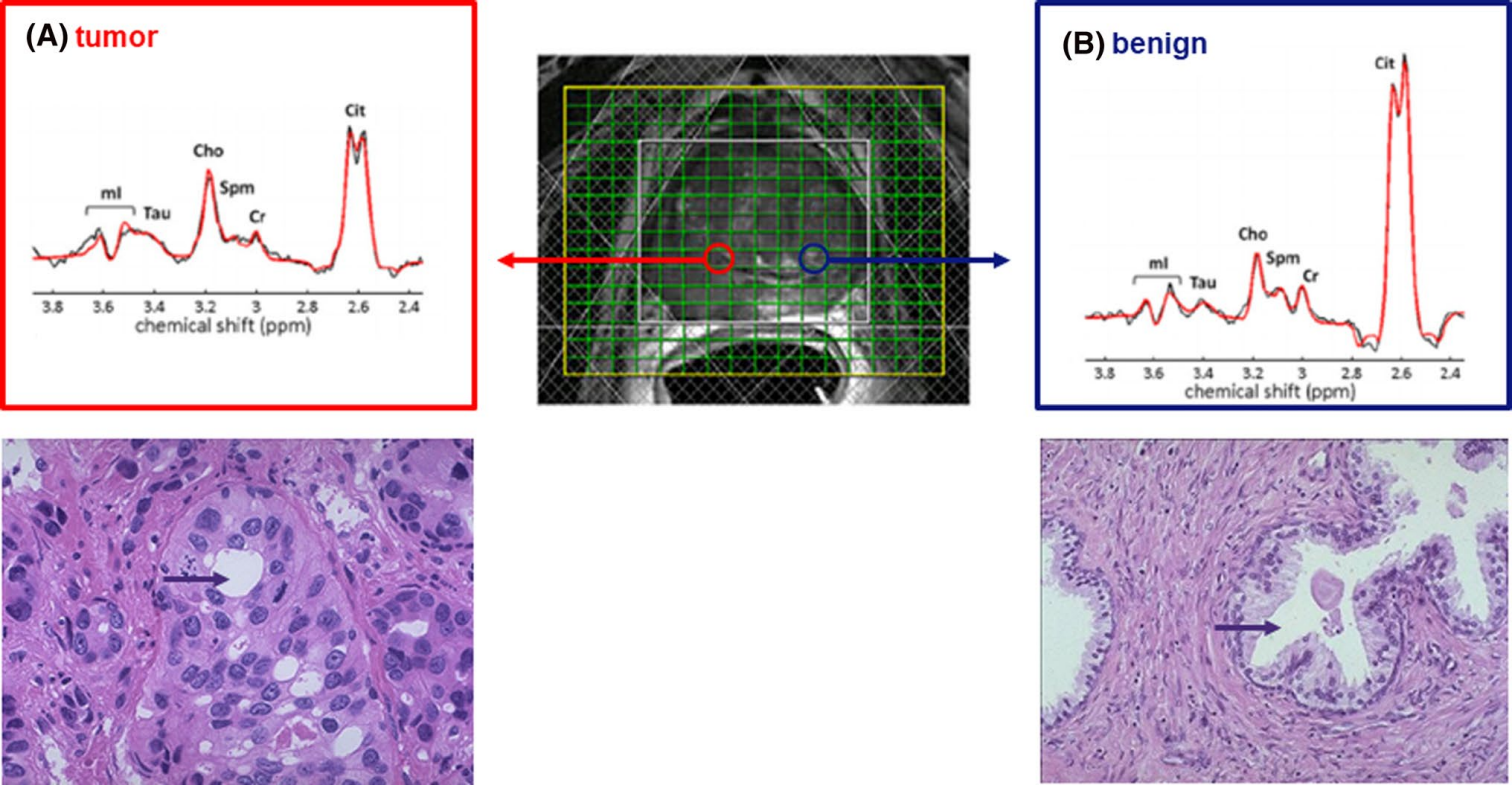
3D MRSI of a patient with histopathology confirmed prostate cancer. The patient was measured at 3 T with an endorectal coil for signal reception and a GOIA-sLASER sequence for VOI (voxel of interest) selection of the prostate. The yellow box indicates the field of view and the white box the VOI. The hatched bars represent the OVS slabs. For further MR measurement details see [28]. On the T2w MR image with the MRSI voxel grid, the location of a tumor voxel (**A**) and a benign voxel (**B**) are indicated with circles to better represent their actual shape which is spherical due to the point spread function. Representative MR spectra from tumor tissue and benign tissue are shown in panel A and B, respectively, illustrating decreased citrate and spermine and increased choline signals in the tumor lesion. Indicated are the signals for the prostate metabolites: citrate (Cit), choline (Cho), spermine (Spm), creatine (Cr), taurine (Tau) and myo-inositol (mI). Under the panels also histopathology slides are shown, illustrating the reduced luminal space in a cancer lesion in comparison with a healthy tissue (purple arrow)

The dominant metabolite peaks observed in MR spectra acquired from the prostate include those from protons in citrate, choline compounds, and (phospho-)creatine (Fig. 1). Usually signals of polyamines, mostly spermine, can also be detected [20, 27]. Furthermore, because of more recent progress in MRSI acquisition other signals, such as of myo-inositol and taurine, may be observed [28, 29] (Fig. 1). Since these signals and their ratios are used as biomarkers for prostate cancer or prostate abnormalities it is essential to understand their MR properties and biological context. Metabolite resonances in ^1^H MR spectra of the prostate can be separated in those that arise from compounds dominant in the luminal ducts (citrate, myo-inositol, spermine) and those dominant in prostate cells (choline compounds, creatine).

Citrate (Cit) has two methylene groups with proton spins that are strongly coupled. Therefore it appears as a quartet signal in ^1^H MR spectra with large variations in spectral appearances at different magnetic field strengths and pulse sequence timing [28, 30]. At lower field (≤ 3T), the two middle peaks dominate and resonate close together at about 2.6 ppm (Fig. 1). The citrate signal shape and chemical shift also depends on pH [31] and cation concentration [32].

The choline compounds glycerophosphocholine, phosphocholine and free choline (Cho or tCho for total choline) have nine chemically equivalent protons of three methyl groups resonating as a singlet around 3.19 ppm and two methylene groups, resulting in multiplets at 4.05 and 3.50 ppm. In practice, only the nine-proton singlet at ~3.19 ppm is evaluated because the intensity of the multiplets is very low in in-vivo MR spectra of the prostate.

Creatine (Cr) has five non-exchanging protons, a methyl group resonating at 3.03 ppm and a methylene group at 3.93 ppm. The protons in each group are chemically equivalent and uncoupled, resulting in two singlets [33].

Spermine (Spm) contains, next to its amine groups, ten methylene groups that form a weakly coupled spin system. The methylene protons consist of symmetrical pairs, giving a total of four protons resonating at about 1.81 ppm with further groups of four at about 2.11 ppm, 3.13 ppm, 3.12 ppm, and 3.18 ppm. Usually only the signals at 3.12–3.18 ppm are evaluated as the others are suppressed due to lipid signal suppression pulses present in most pulse sequences. The chemical shift of these resonances is sensitive to pH and their shape and intensity may depend on TE [27, 34, 35].

Myo-inositol (mI) is a closed-ring sugar with six coupled protons resulting in a complicated spectroscopic shape with the highest intensity at about 3.5–3.6 ppm [36].

### Metabolites in healthy prostate tissues

The healthy prostate accumulates high levels of Cit (Fig. 1b), in particular in the peripheral zone [37]. This zone consists of layers of glandular epithelial cells surrounding prostatic ducts. Epithelial cells in the prostate highly express the zinc transporter ZIP1 [38]. The resulting high intracellular concentration of zinc inhibits the enzyme aconitase in the tricarboxylic acid (TCA) cycle. This causes a high production of Cit at the cost of TCA cycle related energy production per molecule of glucose [36, 37]. The excess Cit is secreted in the prostatic fluid of the lumen and contributes to favorable conditions for sperm maturation and motility in seminal fluid [41–43]. It has been reported that the Cit levels in normal prostatic fluid may vary considerably between about 10 and more than 300 mM [39, 40, 44, 45]. A mean value of about 100 mM is often assumed [44]. In vivo MRS assessments estimate normal prostate tissue Cit concentrations to be between 30 and 70 mM [29, 46–48]. With about a quarter of peripheral zone tissue volume occupied by ductal luminae this would correspond to Cit levels in prostatic fluid within these luminae between 120 and 280 mM. Similar tissue Cit levels were found in glandular prostate tissues by high-resolution magic angle spinning spectroscopy (HRMAS) [49]. In glandular tissues with benign prostatic hyperplasia (BPH), Cit levels may be higher [40, 47, 48], or lower in stromal BPH with less luminal space [49].

Myo-inositol is another major metabolite in prostatic fluid [45]. In this fluid, it occurs on average at about 6% of the citrate content which would mean that its normal tissue concentration would be 1–4 mM, although HRMAS studies estimate a higher tissue concentration [50]. The high concentration of myo-inositol in prostatic fluid serves multiple functions in male fertility such as in osmoregulation of seminal fluid to enhance sperm motility and in improving sperm mitochondrial function [51].

In healthy prostate tissues, high concentrations of polyamines are present [49] mainly representing spermine [52]. Similar to citrate, they are secreted by specialized ductal cells in the prostate [53], and likewise accumulate in the luminal space [29]. Ornithine decarboxylase is the key, rate limiting, enzyme in the synthesis of polyamines. Apart from their role in proliferation and cell growth, they have various functions in fertility and contribute to the motility of sperm cells [54]. From extrapolation of prostatic fluid, HRMAS and in vivo MRS studies the tissue concentration of spermine is estimated to be 7–18 mM [29, 45, 49]. HRMAS studies indicate that the tissue content of polyamines are lower in stromal tissue and higher in glandular tissue [49]. A linear relationship between the concentration of polyamines (spermine) and citrate has been observed [36, 49, 55] and a transient association between citrate, spermine and zinc and binding of this complex to proteins has been deduced from in vitro T2 relaxation studies [56].

Choline and creatine compounds are present at relatively high concentrations in prostate cells. As these compounds are not detectable, or at very low concentrations, in prostatic fluid [36, 45], their signals observed in vivo must come from prostate tissue, which could be epithelial or stromal cells. From MRS measurements of healthy prostates the Cho tissue levels were estimated to be between 2 and 5 mM and those of Cr between 4 and 9 mM [17, 29, 33, 46, 49]. Creatine occurs at a higher level in stromal tissue, which is in agreement with the presence of smooth muscle in this tissue [57].

Healthy prostate regions, such as the peripheral zone, transition zone, areas next to the urethra, seminal vesicles and anterior fibromuscular stroma, may have different metabolite compositions. E.g., seminal fluid contains relatively high choline/glycerophosphocholine levels which may affect the spectra of voxels close to or overlapping the seminal vesicles [20, 58, 59]. In vivo ^31^P MRSI measurements of the prostate identified phosphocholine rather than glycerophosphocholine as the main phospho-ester in seminal vesicles [60].

### Metabolites in cancerous prostate tissues

In 1963, it was reported for the first time that citrate levels are decreased in prostate cancer tissue [61]. Later this was observed in vivo with ^13^C MR spectroscopy [62], but it gained real interest as a potential diagnostic tool when it was demonstrated that this decrease could be detected with ^1^H MR spectroscopy [63–67] (Fig. 1).

An early event in the development of cancer in the prostate is the downregulation of zinc transporters [38]. At lower zinc levels the inhibition of aconitase is released and consequently TCA cycling is activated and citrate production and secretion is reduced. Glucose produces energy more efficiently, which may be relevant in malignancy and metastasis [68]. Next to reduced citrate secretion into the prostate lumen, cancer growth also causes a reduction in luminal space [69, 70]. Together these contribute to a lower citrate signal in ^1^H MRSI voxels of cancerous tissue.

Together with a lower citrate signal, the intensity of polyamine (spermine) signals are decreased in ^1^H MR spectra of cancerous prostate tissues (Fig. 1) [49, 50, 52, 55, 71]. This may similarly be explained by less spermine synthesis and decreased luminal space. Polyamine metabolism in prostate cancer is different from benign tissue and it may be relevant for disease progression that spermine can inhibit growth of PCa cells [72–74].

Abnormalities in the metabolism of myo-inositol have been documented and implicated in various disease states, including cancer [75]. In prostatic fluid of PCa patients, the concentration of myo-inositol is decreased [45].

Increased signals of lactate, commonly observed for tumors because of high glycolysis (i.e., Warburg effect), are seen in ^1^H MR spectra of PCa tissue in vitro [49, 76], but these signals appear to be under the detection limit (~1.5 mM) of in vivo 3T MRSI of tumor lesions in the prostate recorded at TE = 144 ms [77]. Furthermore, the methyl lactate signal, if present, is easily obscured by signals of lipids resonating at a similar chemical shift or suppressed together with these signals, a common procedure embedded in most acquisition sequences.

In PCa tissues, the levels of choline compounds are increased, mainly due to a higher phosphocholine and glycerophosphocholine content [49]. This involves increased choline transport into tumour cells, increased choline kinase α and phospholipase A2 expression and activity in tumours [78, 79]. Rising choline levels in tumors are often associated with increased cell density and tumour hypoxia [80]. However, in prostate cancer tissue necrosis is rarely observed [9], which indicates that hypoxia, if present, is limited. In many MRS studies, a correlation has been observed between choline levels or choline signal ratios such as Cho/Cr or (Cho+Spm+Cr)/Cit and Gleason score [19, 71, 81–88]. The increased Cho/Cr ratio in high-grade tumors may also reflect decreased creatine, e.g., due to replacement of smooth muscle tissue by tumour cells or to changes in creatine metabolism [89].

Thus altogether, decreased levels of citrate, polyamines (i.e., spermine) and creatine and increased choline compound levels are attractive biomarkers to identify the presence of prostate cancer. However, some features of the prostate and its condition may mimic these changes and need attention in diagnosis. Citrate levels are highest in the normal peripheral zone and lower in areas close to the urethra. If BPH is mainly of stromal origin, this can result in relatively low citrate levels [49]. As described above, seminal vesicles my contain high levels of choline compounds and need to be identified. Inflammation of the prostate, prostatitis, may mimic changes seen in prostate cancer. This ambiguity can be solved, since the Cit/Cho ratio appears to be higher in prostatitis than in tumours [90].

## Hardware

### Field strength

Common clinical MR systems employed for MR spectroscopy examinations have a field strength of 1.5T and 3T. Prostate MRSI at 3T can be performed at a higher SNR than at 1.5T, as illustrated by the two-fold increase in SNR for the inner citrate resonances, which enables MRSI with a higher spatial resolution [91]. Moving to 7T further increases SNR of prostate MRI 1.7- to 2.8-fold [92]. However, at this field it is more problematic to achieve sufficient RF power and field homogeneity in the prostate. These field-specific challenges are described in a separate paper in this issue [93]. At higher spatial resolution, the MRSI matrices to cover the whole prostate increase, which requires more repetitions in traditional 3D phase encode sampling and therefore may result in too long acquisition times for clinical exams, requiring accelerated acquisitions, e.g., with spiral readouts [94–96].

### Endorectal or phased array RF coils

**Fig. 2:**
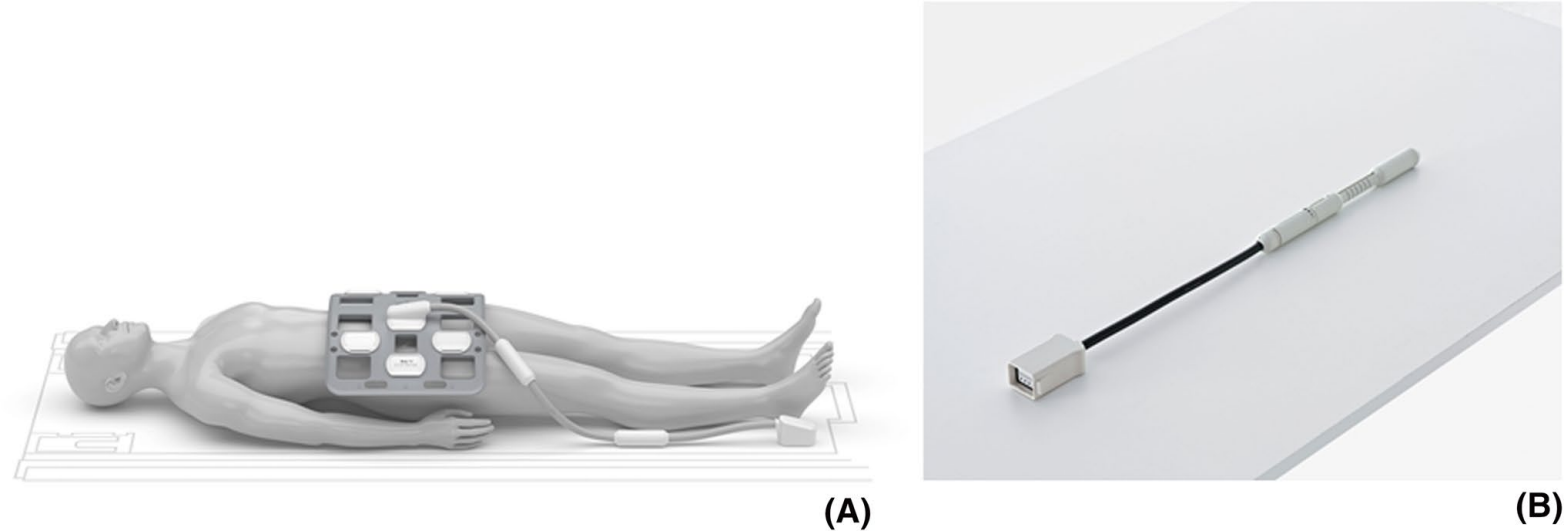
Coils for MR signal reception used for prostate 1H MRI and MRSI. **A** External phased array coil positioned on a patient for prostate MR examinations [233]. **B** Endorectal surface coil for positioning in the rectum [233]

MRSI of the prostate commonly involves spin excitation with a body coil and signal reception with an external multi-channel phased array and/or an endorectal coil (Fig. 2) to receive the MR signal [17, 35, 97]. At 1.5T the use of an endorectal coil (ERC) is recommended if spatial resolutions below 1 cm^3^ are desired with sufficient spectroscopic SNR [20, 98, 99]. Endorectal coils are available in several versions requiring different operating procedures, each with specific benefits and disadvantages for MRS exams [100–105]. Although SNR is lower with phased array coils at 1.5T their use still may have diagnostic value [104].

At 3T MRSI can be performed without an ERC [106], but its use still improves cancer localization compared to using only external phased-array coils [107]. However, a comparison of PCa diagnosis by MRI at 1.5T with ERC and 3T without indicated a similar performance in cancer detection [108]. Although an ERC provides high SNR, especially near the coil, which can be exploited to reduce measurement time or to increase spatial resolution [83, 109] it has several disadvantages in clinical routine compared to phased array coils. For instance, an ERC is costly, its positioning is time consuming, requires experience, is uncomfortable for patients, is associated with signal drop in the coil FOV and causes artefacts [15]. Moreover, using an ERC reduces the SAR limit set by the MR system, which leads to longer acquisition times. Therefore, mpMRI of the prostate is now mostly applied at 3T without ERC, i.e., with external body phased array coils and hence it is relevant to demonstrate that clinical 3D MRSI of the prostate can also be performed without ERC at 3T [81, 88]. Using PRESS for acquisition, the quality of MRSI at 1.5 T with ERC is comparable to that at 3T without ERC, except for voxels located close to this coil, which have a higher SNR [110]. The loss in SNR by performing MRSI at 3T with a phased array coil combination instead of an ERC can be mitigated using LASER type of acquisition sequences [17, 28, 96]. Several studies have demonstrated that in this way MRSI of the prostate can be performed reliably within acceptable time in clinical routine [28, 82, 106, 111].

## Acquisition

### Shimming

An essential first step towards the acquisition of high-quality ^1^H MRSI data is optimization of B_0_ field homogeneity (shimming). This strongly affects spectral quality, since inhomogeneous fields broaden the spectroscopic signals due to faster decay of the apparent transverse magnetization in a voxel. If this causes overlap of signals, it can affect the reliability of their quantification. This may be a challenge for the signals of choline, spermine and creatine, which resonate rather close to each other. Good B_0_ homogeneity is also crucial for effective water and lipid signal suppression, since when the signals are broadened or shifted they may escape the frequency-selective pulses needed for their suppression [20]. The signal broadening effects of field inhomogeneity may be restored by a modulus operation on the FID of water signal unsuppressed ^1^H MRSI [88, 112–114]. A recent post-processing approach to restore field inhomogeneity effects in prostate MRSI is over-discretized reconstruction, which appears to improve lipid signal contamination [115]. Finally, a practical step to improve the B_0_ inhomogeneity is the preparation of the rectum with a cleansing enema and an endorectal gel filling [116].

### Pulse sequences

**Fig. 3:**
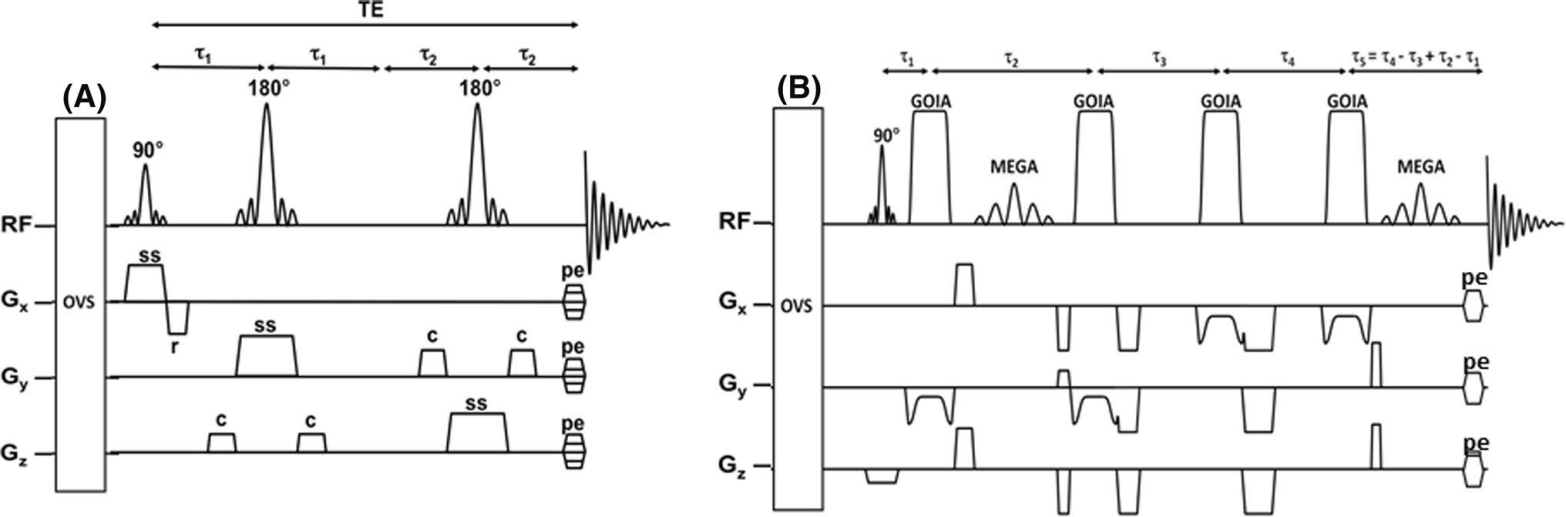
MRS pulse sequences used for volume selection of the prostate. **A** PRESS (point resolved spectroscopy) pulse sequence. OVS = slice selection for outer volume suppression. MEGA: frequency selective pulses for Mescher-Garwood spectroscopic suppression. Acq: acquisition. Interpulse timings are indicated [17]. **B** sLASER pulse sequence. After excitation with a conventional slice selective excitation pulse, the signal is refocused with two pairs of slice-selective low-power adiabatic GOIA refocusing pulses [17]

Initially, localized in vivo MR spectra of the human prostate were obtained using only the field of view of an endorectal coil and single voxel MRS [33, 62, 97]. Volume localization of the prostate for ^1^H MRS was first performed with stimulated echo acquisition mode (STEAM) and point-resolved spectroscopy (PRESS) sequences [117, 118], of which the latter is commonly applied in the clinic (Fig. 3a). Because of the multi-focal and heterogeneous nature of prostate cancer these volume selection methods are now mostly employed in combination with 3D MR spectroscopic imaging methods to cover the whole prostate [19, 20, 119].

More recently, adiabatic pulses were introduced for volume of interest (VOI) localization by adiabatic selective refocusing (LASER) sequences, in which paired adiabatic refocusing pulses are applied for slice selection of a VOI. In the most popular version one pair is replaced by a standard 90^°^ excitation pulse, which is called semi-LASER [28] (Fig. 3b). Adiabatic pulses have better slice profiles, reducing outer volume signal contamination and are less sensitive to RF transmit field inhomogeneities [120, 121]. Also, they have a wide excitation bandwidth, which reduces the chemical shift displacement artefact (CSDA) [122]. However, they are RF power-demanding and need to be applied in pairs to achieve a homogeneous phase distribution over the selected slice, which limits their use [123]. To lower RF power deposition, gradient-modulated offset independent adiabaticity (GOIA) pulses [124] have been implemented for prostate MRSI, since they require less RF power to reach adiabaticity [24]. The application of GOIA-sLASER to prostate MRSI considerably reduces the contamination of spectra with lipid signals of fat surrounding the prostate and hence improves the quality of the spectra and robustness of the measurement [28]. As the T1 relaxation times of the proton spins in prostate metabolites are relatively short (compared to those in the brain), it is possible to select rather short repetition times (TR) so that for instance in a 12x12x10 matrix in 3D MRSI with GOIA-sLASER, the measurement time can be reduced to less than 7 min without an endorectal coil with a nominal voxel resolution of 7 × 7 ×7 mm [81].

To speed up the measurements in Cartesian k-space sampling for MRSI, it is common to apply weighted sampling and Hamming k-space filtering. This also reduces spatial side bands from the point spread function, but enlarges the true voxel sizes [28, 96] To still keep the intended voxel size within limits, larger sampling matrices and field of views (FOV) can be selected, which however may considerably increase measurement times in the case of large prostates. Therefore, to be more flexible, it is advantage to use rapid k-space sampling schemes such as in EPSI or spiral SI acquisition [96, 119, 125].

Also sequences with spectral–spatial-selective pulses were developed that fully excite metabolite spins in the prostate, but not those of peri-prostatic lipids and water [91, 126]. These sequences successfully have been applied in prostate MRSI at 1.5T, 3T and 7T [35, 91, 126], but are sensitive to B_0_ inhomogeneities.

Although MR spectra of the prostate can be acquired by MRSI with fairly resolved signals of metabolites (see Fig. 1) often there is more overlap, in particular between Cho, Spm and Cr and with lipid signals. Therefore, specially designed pulse sequences have been developed to selectively detect Spm or Cit signals [127, 128]. Separation of signals in complete MR spectra of the prostate can be enhanced by J-resolved and correlated spectroscopy which disperses the overlapping resonances into a second dimension, reducing congestion and increasing metabolite specificity, such as for Cho, Spm and Cr and in the detection of glutamate/glutamine and other resonances[129–133]. As these two-dimensional methods are time consuming their MRSI localized versions became too long for clinical applications. This problem was solved by employing readouts with EPSI and compressed sensing (CS), in which signals can be recovered from an acquisition that uses fewer samples than required by Nyquist–Shannon [134, 135]. Currently, for the prostate, these technically advanced methods still have only been applied to human prostate in 2D MRSI mode with relatively large voxel sizes.

### Echo time selection

For spin-echo type localization sequences commonly the shortest possible echo time (TE) is selected to acquire as much signal as possible minimizing T2 relaxation losses. However, in most prostate MRS(I) experiments, a longer TE is chosen to decrease nuisance signals such as of lipids. The signal of the strongly coupled spin system of citrate is dominating in prostate MR spectra and shows considerable variations as a function of interpulse timing (including TE). Therefore, for each pulse sequence timing and field strength a TE is selected with a high citrate absorption signal intensity [30, 136–139]. Typical TE’s for PRESS volume selection at 1.5T are 120–130 ms, at 3T 85–145 ms [140] and at 7T 71-142 ms [35]. For semi-LASER, at 3T an optimal TE of about 85 ms was selected [28] and at 7T of 56 ms [141]. However, successful 2D MRSI of the prostate with TE’s as short as about 30 ms has been performed [29, 142].

### Movement artifacts

Prostate MR measurements may suffer from movement artifacts, due to the location of the prostate near the bowels and relatively long acquisition times. In patients several approaches are available to minimize these artifacts. To limit bowel movement preparation techniques can be performed, including the use of anti-peristaltic drugs, e.g., glucagon or butylscopolamine bromide and the application of microenema to evacuate the rectum if necessary. Dietary restrictions, where the patients are instructed to fast 6h prior to the exam and consume water solely, even though widely applied, do not appear to provide a significant benefit in data acquisition [143]. However, no study specifically addressing the value of bowel preparation in prostate MRSI has been reported. Potential acquisition techniques to reduce motion artifacts include the application of a navigator [144] or to apply rapid acquisition methods, such as spiral readouts starting at the center of the k-space to correct for motion induced phase variations [96, 145]. Finally, in water signal unsuppressed MRSI the water signal can be used to mitigate movement artefacts [112].

### Lipids and water signal suppression

As the prostate is embedded in lipid tissue substantial contamination by lipid signals may occur in MR spectra of prostate voxels, close to the resonances of interest (i.e., of citrate), for instance if the selected VOI overlaps with lipid tissue, by B_0_ inhomogeneity or by bad VOI selection causing signal bleeding from “lipid” voxels at the VOI edges to other voxels by the point spread function. Pulse sequences with bad VOI selection are a main reason for low quality MRSI data and ultimately hamper the correct quantification of metabolite signals. To prevent lipid signal contamination in prostate spectra several techniques have been used, including outer volume saturation (OVS), additional pulses for lipid and water signal suppression, spectral–spatial-selective pulses, better VOI selection (see section “Pulse sequences”), k-space apodization [17], the use of FID modulus [112, 113] and over-discretized reconstruction [115].

It is common to apply outer volume saturation (OVS) bands, positioned around the prostate to reduce extraprostatic lipid signals (Fig.1). All spins in these bands are excited and then dephased by crusher gradients. OVS pulses were developed to compensate for poor edge profiles, B_1_ field inhomogeneity and chemical shift errors, such as very selective saturation (VSS) pulses with reduced B_1_ and T_1_ dependency [146]. The OVS slabs are usually placed manually, which is subjective and time-consuming, and limits the number of slabs to be placed. Therefore, automated algorithms have been developed, to optimize orientation, timing and flip angle setting of the VSS pulses following the shape of the prostate [147, 148]. Also, supervised 3D fully convolutional networks have been developed, for automatic prostate MRI segmentation [149], that can be extended to MRSI. As OVS selection may affect spectra of voxels at the edge of the prostate it is recommended to keep the number of slabs low and rely on the proper implementation of other options for lipid signal suppression (see above).

A widely applied approach is spectroscopic signal suppression with double band-selective inversion with gradient dephasing (BASING) [150, 151] or Mescher–Garwood (MEGA) [152], in which dual-frequency pulses surrounded by crusher gradients, selectively invert and dephase both the lipid and water signals.

Signal contamination between neighboring voxels, due to the side-lobes of the spatial response function (SRF) can be significant when peri-prostatic areas with high lipids are included in the VOI. These side-bands are commonly attenuated with a Hamming apodization filter in k-space [153]. Combined with weighted elliptical k-space sampling this results in a considerably shorter acquisition time [154], which however increases voxel size, and thus SNR, but reduces spatial resolution [96, 155]. Another significant method to prevent lipid contamination is the application of more accurate localization sequences (see above Pulse sequences [77, 156]).

## Processing and interpretation of ^1^H MRSI data

Current protocols to process MRSI data require the performance of multiple steps, like quality control of spectra, localization of cancer suspicious voxels with reference to MR images [86, 138, 157], and judging a spectrum as ‘suspicious of tumour’ or not [158, 159]. Proceeding through all these steps manually is time consuming and requires significant effort, since thousands of spectra are acquired from each patient, which demands time and experience of the users. Therefore, automation in data processing is essential in clinical routine.

The following paragraphs discuss processing steps for ^1^H MRSI data of the prostate and how these steps can be performed in a reproducible, automatic way.

### Signal preparation

In case of data acquisition with multiple receive coils, the signals of these coils have to be properly combined and arranged in k-space, which may require specific methods for MRSI applications [160]. In case of signal reception with an endorectal coil B1 inhomogeneity may have to be corrected [161]. For MRSI usually apodization filters are applied, to improve the shape of the SRF, and the matrix of the data is zero filled, to facilitate a better localization of spectra, but causing smaller voxel sizes than the acquired true voxel size.

### Phase and frequency corrections

After Fourier transformation signal frequency and phase errors may have to be corrected. Numerous algorithms exist for automated phasing and frequency alignment, e.g., [162–164]. Phase correction may be avoided by working on magnitude spectra, but this broadens the line width, reducing spectroscopic resolution. If water signal unsuppressed data are acquired an automatic way for phase and frequency alignment is by computing the modulus of the time-domain MRS signal [113, 165]. Furthermore, principal component analysis can be used to calculate phase and frequency deviations and correct them to achieve an iterative improvement in similarity across all spectra [166, 167]. This has been adapted to automatically correct ^1^H MRSI data of patients with prostate cancer [163].

### Baseline correction

In MR spectra, a baseline can be present under the metabolite signals due to broad signals of macromolecules, insufficient suppression of water and lipid signals or first order phase roll. This baseline has to be taken into account for a proper quantification of metabolite signals. As most prostate MR spectra are acquired with spin-echo type of pulse sequences, acquisition can start at the center of the echo, minimizing first-order phase errors. Fortunately, in contrast to MR spectra of the brain, there is no significant contribution of macromolecular signals to the baseline in MR spectra of the prostate down to echo times of 32 ms [18, 29]. Baseline corrections may be needed if the tails of broad water or lipid signals stretch out into the spectral region of interest (about 2.3–4 ppm). These contaminating signals occur less often with adiabatic volume selection such as applied in sLASER than with PRESS using standard refocusing pulses [28] and are also attenuated at TE’s > 100 ms.

Most quantification software includes a baseline component within the FID or spectrum model. This baseline is often assumed to be a smoothly varying line that can be modelled with splines [168–170] or estimated by smoothing the spectrum [157, 171, 172]. The baseline is corrected together with metabolite fitting by iterative optimisation. Baseline correction without any metabolite estimation is suitable for pattern recognition analysis. This can be achieved by filtering firstly water and lipid signals [173, 174], or by fitting a lipid peak to the spectrum and subtracting it from the region of overlap with the citrate resonance [164]. Accurate baseline correction is important if signal integration is used for quantification. This may require additional quality control as even small variations from zero at the base of a metabolite peak can generate large errors in its total area estimate [138, 175].

### Lineshape modelling and linewidth

Field inhomogeneities within ^1^H MRSI voxels, eddy currents and bad shimming result in non-Lorentzian lineshapes with variable line widths. In quantification algorithms a variable to describe this lineshape can be included, assumed to be the same for each peak within one spectrum, or it can use a fixed model shape, like Gaussian, allowing some variation of the linewidth [86, 176, 177]. Numeric integration can be affected negatively by changes in linewidth if the width of the chemical shift region for integration is fixed [150]. An increased linewidth leads to less of the peak within the integration window and an artefactual underestimation of the resonance area. The effects of varying linewidth need to be checked in a quality control step prior to integration of metabolite quantities or, alternatively, linewidths can be adapted according to the quality of each voxel shim [178].

### Spectroscopic quality control

As SNR in MRSI is limited the technique is prone to artefacts and therefore spectroscopic quality control is an essential step in the clinical pipeline. Quality control can be performed qualitatively by experts, but this is time consuming and not objective. Therefore, automated methods have developed. In one method, a nonlinear classifier of magnitude spectra was used to determine whether spectra were of suitable quality [174]. The classifier was trained on data that expert spectroscopists had graded to be of acceptable or unacceptable quality. Another approach applied feature extraction of the real part of the spectra to reduce it to a small number of scores [179]. The feature extraction is a linear fit of components generated by independent component analysis of spectra from a training set of similarly acquired ^1^H MRSI data. A nonlinear classifier was then applied to these scores rather than the full spectra. The quality control of the spectra was implemented with feature extraction of test-set spectra. Finally, a simple spectroscopic quality control of prostate MR spectra has been proposed in which a ratio of components contributing to bad spectra (noise, lipid signals) and those contributing to good quality spectra (Cit, Cho signals) is used as an index to exclude bad quality prostate MR spectra [180].

Applying a set of rules to judge spectroscopic quality in 3D MRSI of patients by an expert panel showed that using a PRESS volume selection sequence at 1.5T resulted in 33% of voxels with bad quality spectra, while this number was 17% for PRESS applied at 3T and 11% for sLASER applied at 3T without using an endorectal coil. This indicates that PRESS 3D MRSI applied at 1.5T was not well suited for clinical routine, in contrast to the currently used sLASER 3D MRSI [179, 180].

### Spectroscopic evaluation

**Fig. 4:**
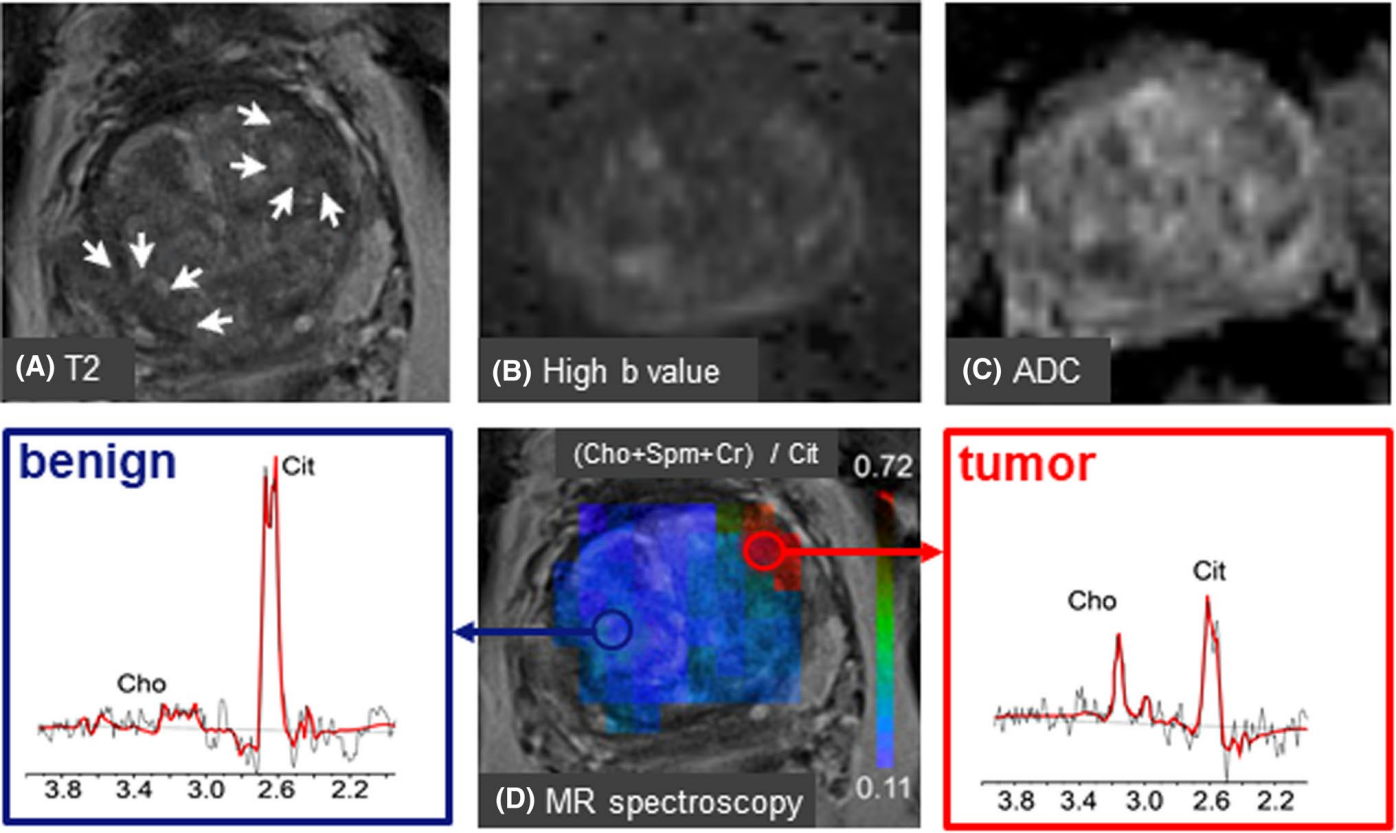
3D MRSI identifies correct tumor lesion after ambiguous T2 and DWI MRI in the mpMRI examination of a patient with serum PSA of 4.3 ng/ml. The MR investigations were performed at 3 T with a phased array coil for signal reception. For details see [111]. **A** Transversal T2 w MRI shows at least two locations with low signal intensity suspicious for cancer in the left and right side of the transition zone (TZ) of the prostate (white arrows). **B** Both these locations have high intensities in high *b* value maps suggesting the presence of tumor tissue. **C** This assignment seems to be confirmed by ADC maps with low intensities at both positions. **D** A metabolite ratio map obtained from 3D MRSI with a GOIA-sLASER sequence shows a hot spot for only the left TZ location. An MR spectrum obtained from this location shows a low citrate and increased choline signal compared to the right TZ location. This identifies the left TZ lesion as cancer tissue and the right TZ lesion as benign. This was confirmed by histopathology of biopsies from both locations, which identified the left TZ location as a low-risk cancer lesion and the right TZ location as benign. *Cho* choline; *Cit* citrate

Metabolite signals in prostate MR spectra can be evaluated qualitatively, for instance, by visually inspecting signal intensity decreases of Cit and Spm and a signal increase of Cho as spectroscopic signatures to identify cancer lesions. However, as this is subjective it is preferred to employ quantitative methods, for instance by peak fitting, either in time or frequency domain. Most MR systems have software to determine peak areas, but it is also possible to export MRS data to specialized spectroscopic processing packages, such as LCModel [170, 181], jMRUI [182, 183] and Tarquin [184]. Because of the use of endorectal coils with an inhomogeneous receive B_1_ field by which signal intensity of the coil drops towards the ventral parts of the prostate, it has become custom to calculate signal ratios, avoiding intrinsic spatial variations in signal intensity. Because it is not always feasible to resolve Cho from Cr and Spm signals, in particular at 1.5T and with PRESS sequences, the most often used ratios are (Cho+Cr)/Cit and (Cho+Spm +Cr)/Cit [18]. With better resolution, e.g., at 3T, it is common to also use Cho/Cr [81] and Cho/Cit ratios. For the localization and characterization of cancer lesions, threshold values for these ratios are determined and ratio maps constructed (Fig. 4c). With phased array coils, which have a homogeneous B_1_ receive field within the prostate, it is possible to evaluate and map the signals of individual metabolites [81].

Absolute tissue concentrations of metabolites (*Abs_con*) can be obtained using the signal of internal water as a reference. As this requires an additional time consuming MRSI measurement without water signal suppression, it is not commonly applied [33, 47]. However, we recently demonstrated that with water signal unsuppressed MRSI of the prostate it is possible to use the intravoxel water signal as a reference to obtain *Abs_con* values. This signal was extracted from the spectra either with water-signal modeling [185] or with tensor-based Blind Source Separation [114].

*Abs_con* is derived from the metabolite signal relative to that of water (*S*_rel_), applying correction factors for T_1_ and T_2_ relaxation of both metabolite (*T*_*1met*_*, T*_*2met*_) and water reference (*T*_*1ref*_*, T*_*2ref*_) according to Equation 1. Relevant T_1_ and T_2_ relaxation times for prostate compounds are presented in Table 1. *Abs_con* is then calculated from the water concentration of prostate tissue *wcon*=40.2 mM, assuming a prostate tissue water content of 39.4 mM/ gr wet weight and a tissue density of 1.02 kg/l [47, 186].

**Table 1:** In-vivo T1 and T2 relaxation times of the prostate metabolites of interest

	Metabolite	T_1_ (s)	T_2_ (s)
1.5 T	Citrate [33, 47 ]	0.34 ± 0.04	0.17 ± 0.02 (PZ)
			0.12 ± 0.03 (TZ)
	Choline [33]	0.84 ± 0.09	0.23 ± 0.06
	Creatine [33]	0.86 ± 0.1	0.21 ± 0.1
3 T	Citrate [30]	0.47 ± 0.14	0.17 ± 0.05
	Choline [30]	1.1 ± 0.4	0.22 ± 0.09
	Creatine [46]	1.13 ± 0.15	0.19 ± 0.02
	Spermine [29]		0.053 ± 0.016
	Myo-inositol [29]		0.090 ± 0.048
	Water-healthy tissue(PZ)	1.756 [234]	0.142 ± 0.024[235]
	Water- cancer tissue(PZ)	1.301 [234]	0.109 ± 0.020[235]

1 $$Ab{s}_{con}=Srel\frac{\left[1-{e}^{\left(-\frac{TR}{{T}_{1, ref}}\right)}\right]\left[{e}^{\left(-\frac{TE}{{T}_{2, ref}}\right)}\right]}{\left[1-{e}^{\left(-\frac{TR}{{T}_{1, met}}\right)}\right]\left[{e}^{\left(-\frac{TE}{{T}_{2, met}}\right)}\right]}wcon$$

In the calculations for prostates with cancer lesions, it was assumed that T1 and T2 values of metabolite spins and *wcon* do not differ between tumor and cancer tissue, while different relaxation values for water spins were implemented (Table 1). Indeed, it is known that tissue water content is rather homogenous over the prostate and does not differ between tumor and benign tissue. Because both T2 and T1 values of water spins in tumors decrease the relaxation correction factor for these spins in benign and tumor tissue are similar for common Tr values, implying that spatial maps of *Abs_con* obtained with this correction factor directly reflect differences in metabolite content between tumor and benign tissue [185].

### Pattern recognition

An alternative quantitative approach to distinguish tumour from benign tissue is by pattern recognition. The process is based on feature extraction, reducing the dimensionality of the MRS data so that useful information is retained, while irrelevant components and noise are removed. Then, these features are classified, separating the data into the anticipated groups. In pattern recognition, spectra are classified without using a biological model and are trained on feature-extracted data to establish which features are important for discrimination. Thus, maps of tumour presence or aggressiveness can be created from the raw data. For instance, it was reported that using nonlinear classifiers on feature extracted data can better separate malignant from benign prostate tissue than using quantification algorithms [187]. Also, a partial least squares regression approach of magnitude spectra achieved similar classifications of benign and tumorous tissues as experts [174].

## ^1^H MRSI in clinical applications

In clinical applications, 3D ^1^H MRSI of the prostate is always applied within a mpMRI approach including T2 weighted MRI and DWI and often DCE MRI so that anatomical and other functional information is also available [12]. Within this context, MRSI has been demonstrated to have significant clinical value in most steps towards PCa diagnostics, including in-vivo detection and localization of tumor lesions, tumor staging, determination of tumour aggressiveness and therapeutic planning and evaluation [19, 20, 86, 157, 188, 189].

### Detection and localization of prostate cancer lesions

For patients with clinical signs suspicious for the presence of PCa the performance of a mpMRI exam is recommended to detect if significant cancer is present. This exam is also important to identify the locations of cancer lesions in the prostate as this information is relevant for staging, TRUS- or MR-guided biopsies, preparation of a radical prostatectomy and guidance for focal therapies [12]. The ability of ^1^H MRSI to detect and localize cancer tissue in the prostate has been demonstrated by numerous studies [17, 19, 20], including multi-center trials [87, 138].

Several studies showed that the sensitivity of ^1^H MRSI to identify cancer lesions increases with the GS, for instance at 1.5T a sensitivity increase from 44 for 3 + 3 to 89% for ≥ 4 + 4 GS lesions was recorded [157] and the sensitivity for the detection of high-grade was greater than of low grade disease ((92.7 vs. 67.6%) [190]. Most likely the smaller volume of the low-grade lesions contributed to this lower detection sensitivity by ^1^H MRSI at 1.5T due to a partial volume effect involving signal mixing with normal prostate tissue in the voxels. However, the better sensitivity in detecting high-grade tumors might be used to exclude patients with these tumors from active surveillance [190].

In a prospective multi-site study, it also appeared to be difficult to detect low grade, small volume tumors [159]. Together with a low overall spectroscopic quality in this and other studies this raised questions about the role of MRSI in detection of tumors in clinical routine [191]. However, most clinical studies until recently were performed with standard PRESS localization at 1.5T with endorectal coil or with standard PRESS at 3T without endorectal coil. As outlined above our quality assessment demonstrated that a substantial fraction of MR spectra measured by MRSI under these conditions have insufficient quality and hence are not suitable for routine clinical applications (see above).

As also outlined above the robustness of MRSI can be improved using LASER type of sequences. In recent studies, it was demonstrated that employing a GOIA-sLASER sequence at TE=88 ms and only a phase array coil for acquisition and using a support vector machine model analyzing several metabolite ratios, it is possible to discriminate tumor lesions, of low up to high GS, from normal prostate tissue in the transition zone with high accuracies of 96% [82]. Even better results were obtained in an mpMRI exam, in which MRSI was combined with T2 weighted and diffusion weighted MRI [111]. The complementary role of MRSI in tumor localization by mpMRI is illustrated by the case shown in Fig. 4, in which ambiguity about the tumor location after T2 and DWI MRI could be solved by adding MRSI data. Similar results on the value of MRSI in the separation of tumor lesions and normal prostate tissue by mpMRI were reported for examinations performed with an endorectal coil at 3T using an MLEV-PRESS sequence for volume selection at TE=85 ms [83].

### Tumor aggressiveness

Because most prostate cancers grow slowly and are not life threatening it is a major clinical problem to identify aggressive tumors among indolent ones to avoid overtreatment. Low aggressive tumors may be selected for active surveillance instead of surgery or another drastic treatment. For instance a clinically non-significant tumor nodule is organ-confined with no GS higher than 3 and with a volume smaller than 1.3 cm^3^ [192]. As Gleason scoring from biopsies suffers from sampling errors it would be very important if functional imaging could discriminate between aggressive and indolent tumors and predict progression of the latter to aggressive variants.

In general, a correlation is found between GS and metabolite ratios, but the overlap of ratio values between risk groups precluded to only use these to assign individual cases to certain risk groups, although MRSI appears to perform better than DWI in the transition zone, while DWI performs better in the peripheral zone [193]. However, in some studies reasonable separations between low risk and higher risk cancer lesions were obtained when metabolite ratios were combined with T2 w MRI, DWI or DCE parameter values [194]. By adding MRI and MRSI measures to a clinical nomogram, it was possible to identify significant cancer at an AUC of 0.85 [195]. In a study using a GOIA-sLASER sequence at 3T with TE=88ms to measure metabolites and a support vector machine analysis it was possible to separate low risk cancer lesions from high risk in the transition zone in a combination of ADC, Ktrans and metabolite ratios at an AUC of 0.86, while AUC was only 0.64 if ADC was combined with Ktrans [111].

## Challenges and future directions

Although mpMRI is widely applied to men with elevated PSA to detect clinically significant PCa a recent Cochrane meta-analysis revealed that the pooled specificity of mpMRI was only 37%, despite a high sensitivity [22]. Moreover, mpMRI suffers from low inter-reader reproducibility [21, 22]. This clearly indicates that additional approaches are needed to improve PCa diagnosis. Currently, the role of DCE MRI as a useful part of an mpMRI exam is under discussion because of the relatively small contribution to diagnosis and time and effort to prepare the patient for iv injection and costs [196, 197]. For these reasons, the inclusion of ^1^H MRSI in mpMRI may be considered as a valid alternative, even if only selected to assist in equivocal cases. For MRSI to be included in routine clinical workflow, some of the innovations described in this review are indispensable and still further innovations would be helpful to improve robustness, speed, spatial resolution and data processing. Some recent new technological developments are anticipated to provide these improvements for clinical prostate MRSI.

As described above the replacement of standard PRESS volume selection by sLASER is a major step forward and MRSI with sLASER in a mpMRI approach using machine learning yielded excellent results in the detection and localization of cancer lesions in the prostate and determination of the aggressiveness of the disease [111]. Another potentially important new acquisition option is sLASER volume selection in MRSI without water signal suppression. This may open new opportunities in the diagnostics of prostate diseases as it simultaneously provides information of both water and metabolite signals. Moreover, the water signal can be used to correct for line shape artefacts, as a reference to estimate absolute metabolite levels, and to accurately combine signals of multiple coil elements with different sensitivity profiles. We recently demonstrated for water-unsuppressed MRSI of the prostate that it is possible to remove the water signal and its artefacts in postprocessing and use this signal for referencing purposes [88]. Because no additional MRSI acquisition is required it can be performed within a clinical exam [88, 114].

New options for accelerated 3D MRSI acquisition of the brain, such as by SPICE (SPectroscopic Imaging by exploiting spatiospectral CorrElation), which makes high-resolution metabolic imaging possible by incorporating prior information and field inhomogeneity corrections, or by compressed sensing and low rank reconstruction, may be of interest for prostate applications as well [198–200].

In the recent past, the use of an endorectal coil was considered inevitable for MRI and MRS of the prostate to obtain data with sufficient SNR and spatial resolution. However, upon increasing field strength from 1.5 to 3 T it has become common to perform MRI without an ERC as it turned out that clinical performance was similar, patient discomfort is relieved, patient preparation time is shortened and avoids the cost of an ERC. Therefore, it is unavoidable that clinical MRS of the prostate also needs to be performed without an ERC, even though it has been advocated that detection of small lesions may require such a coil [83]. In several studies, it was shown that MRSI of the prostate with a phased array coil is feasible at 3T, moreover the application of an sLASER sequence at a TE of about 85 ms substantially improved SNR allowing to perform MRSI of the prostate with a high reliability [28, 201].

Local B_0_ field inhomogeneities, caused by magnetic susceptibility differences between prostate tissues, may affect MRSI quality. The most severe inhomogeneity occurs at air-tissue transitions such as at the rectum. Commercial MR systems can correct for B_0_ inhomogeneities with shim coils, but these usually are not able to handle multiple boundaries with strong susceptibility transitions [18]. To improve this situation, a range of dedicated technical solutions have been developed, mostly for brain applications [202–206]. It would be of great interest to explore some of these for prostate applications in particular as diffusion MRI of the prostate, the mainstay in PCa diagnosis, also suffers from problems with susceptibility transitions. Local B_o_ inhomogeneities can also be corrected in postprocessing such as by over-discretized reconstruction, which was demonstrated to improve signal localization in prostate ^1^H MRSI, and therefore reduced lipid signal contamination [115].

Currently, there is a rapid development to introduce artificial intelligence (AI) methods for improved acquisition, processing and interpretation of MRI [207]. Several AI methods have already been applied to MRSI of the brain to estimate tissue concentration of metabolites [208, 209], to enhance spatial resolution of MRSI [210] and to asses MRSI spectral quality and filter artifacts [211, 212]. Specifically for prostate MRSI, the application of AI may also be of interest for optimal OVS to suppress lipid signals [213–215].

With SNR being critical in clinical MRSI, the application of denoising methods has attracted much attention recently. A popular approach is low-rank denoising, which is performed by arranging the measured data in matrix forms (i.e., Casorati and Hankel) and applying low-rank approximations by singular value decomposition (SVD). The method can effectively denoise MRSI data over a wide range of SNR values while preserving spatial-spectral features [216, 217]. Also, methodologies incorporating pre-learnt spectral basis sets and spatial priors in low-rank approximation methods [218] and principal components analyses (PCA) [219] may be of interest for prostate ^1^H MRSI, and also multi-nuclear (^31^P, ^13^C) MRSI applications [220].

All these new methods may significantly improve the effective SNR of multidimensional ^1^H MRS. As this can be translated to faster measurements and improved spatial resolution, this can help to overcome some final hurdles in prostate MRSI towards clinical application, in particular if data processing steps are automated, ideally in an mpMRI integrated AI algorithm.

A recent multicenter study evaluating PIRADS scoring of mpMRI with an AI-based attention mapping system only showed marginal improvements in PCa detection [221]. One reason may be that the information content of mpMRI data is simply not sufficient for AI methods and that additional imaging information is needed, which may include that of MRSI. For the classification of MR spectra from voxels to PCa detection, lesion location and grades, machine learning approaches have been applied [82, 94, 111, 174, 187, 222–225], essentially using histopathological analysis of whole mount prostatectomy or of image-guided acquired biopsy specimens as gold standard. Training and validation of these AI methods is not trivial as it requires a substantial amount of annotated MRSI data, although convolutional neuronal networks dedicated to limited MRSI data sets have been developed to predict brain tumor grades and other brain diseases [226]. Sharing data may be a solution to this problem [227]. A remaining issue in validation of all these methods is accurate matching of MR(S)I data to histopathological standards of reference (either MR-guided biopsies or whole-mount section histopathology of resected prostates).

Current prostate diagnostics by mpMRI relies on a histopathological tumor classification which has been developed more than 40 years ago [5]. In the diagnosis of brain tumors classical histopathology has been largely replaced by a classification based on molecular markers to discriminate among tumor subtypes [228]. This is now also practiced in MRI diagnoses of these tumors and has opened new possibilities for MRSI to assist in precision diagnosis, which is most obvious in the identification of IDH1 mutations by 2-hydroxyglutamate [229]. Although no such metabolite has been detected yet for PCa, specific metabolite patterns may be related to genetic molecular or liquid biopsy markers of clinical significance [230] and used to assist in a more personalized diagnosis and treatment.

Most MR biomarkers to identify and characterize PCa rely on a decrease of signal intensity (T2, DWI (ADC), citrate, spermine). This is not ideal as it may not be specific enough. Imaging methods in which signals increase because of cancer presence such as in choline MR imaging and PET imaging would be better suited. Adding ^68^Ga PSMA PET imaging to mpMRI in a PET/MR system has been proposed to improve the low specificity of mpMRI in the detection of clinically significant prostate cancer [231]. Although expensive PCa diagnosis by PET imaging is in full development [232] it would be of interest to compare its performance to mpMRI including ^1^H MRSI. If MRSI could replace PET in this approach, it would prevent a large overload in examination costs and patient burden.
